# HIV Stigma Reduction for Health Facility Staff: Development of a Blended- Learning Intervention

**DOI:** 10.3389/fpubh.2018.00165

**Published:** 2018-06-21

**Authors:** Laura Nyblade, Krishnamachari Srinivasan, Amanda Mazur, Tony Raj, Divya S. Patil, Dhinagaran Devadass, Kedar Radhakrishna, Maria L. Ekstrand

**Affiliations:** ^1^Global Health Division, International Development Group, RTI International, Washington, DC, United States; ^2^St. Johns Research Institute, Bangalore, India; ^3^Department of Medicine, Center for AIDS Prevention Studies, San Francisco, CA, United States

**Keywords:** HIV, stigma, mHealth, education, health care workers, intervention development

## Abstract

**Introduction:** The effect of stigma on health and health inequity is increasingly recognized. While many medical conditions trigger stigmatization, the negative effects of HIV stigma are particularly well documented. HIV stigma undermines access, uptake, and adherence to both HIV prevention and treatment. People living with HIV face stigma in all aspects of their daily lives; however, stigma in the health system is particularly detrimental. A key component for health facility stigma-reduction interventions is participatory training of staff, often through several days of in-person training. Though this approach shows promise, it is time intensive and poses challenges for busy health facilities. In response, the DriSti study has developed a brief blended-learning approach to stigma reduction in Karnataka State, India. This paper describes the process and final content of the intervention development. The intervention is currently being tested. Final evaluation results will be published upon study completion.

**Methods:** Grounded in behavior change strategies based on social cognitive theory principles that stress the importance of combining interpersonal interactions with specific strategies that promote behavior change, we used a three-phase approach to intervention development: (1) content planning—review of existing participatory stigma-reduction training activities; (2) story boarding—script development and tablet content production; and (3) pilot testing of tablet and in-person session materials.

**Results:** The final intervention curriculum consists of three sessions. Two initial self-administered tablet sessions focus on stigma awareness, attitudes, fears of HIV transmission, and use of standard precautions. The third small group session covers the same material but includes skill building through role-play and testimony by a person living with HIV. A study team member administers the tablet sessions, explains the process, and is present throughout to answer questions.

**Conclusion:** This paper describes the theoretical underpinning and process of developing the blended-learning curriculum content, and practical lessons learned.The approach covers three key drivers of HIV stigma—stigma awareness, fear of HIV transmission, and attitudes. Developing video content for the self-directed learning is complex, requires a diverse set of people and skills, and presents unexpected opportunities for stigma reduction. Co-facilitation of the in-person session by someone living with HIV is a critical component.

## Introduction

Stigma is increasingly being recognized as a fundamental determinant of health and health inequity, alongside factors like socio-economic status and gender (1). While there are many medical conditions that trigger stigmatization, the negative effects of HIV stigma are particularly well documented. HIV stigma undermines access to, uptake of, and adherence to HIV prevention and treatment services (2–6). While HIV stigma occurs across all spheres of life—from partners to extended family, from neighbors to co-workers, in the workplace and in schools—when it occurs in the health system it is particularly detrimental to health, whether for prevention or treatment (7). The ubiquitous presence of stigma in health care settings across the globe is well documented (8–11). These range from more visible forms like outright denial of care (9) and verbal abuse (12) to more subtle forms like lower standards of care (12), senior staff passing off care to junior staff (10), gossip, demeaning body language (13), and longer wait times (14). In India, studies have found high rates of HIV-related stigma among health care professionals, which may result in endorsing coercive policies, breaches in confidentiality, and differential treatment based on HIV status. Health care facilities have been known to engage in such discriminatory activities as burning of bed linens used by clients living with HIV, billing clients living with HIV for the cost of infection control supplies, and using double gloves or gloves for all interactions with these clients (14–16).

Growing recognition of the urgency to respond to HIV stigma within the health system is reflected in a recent call and initiative launched by the Joint United Nations Programme on AIDS and the World Health Organization (17). While not yet implemented at any scale, evidence on how to intervene to reduce HIV stigma in health facilities is growing (18–23). These interventions are grounded in behavior change theory and focus on creating space for contact between those perpetrating stigma and the stigmatized (contact strategies) (24–28), fostering empathy (29–32) and building the knowledge and skills necessary to change stigmatizing behavior (27, 33–38). This evidence, coupled with years of intervention efforts, has established basic principles for stigma-reduction programming, which can be designed to operate on what are often referred to as the immediately actionable drivers of stigma (11, 19, 21, 23, 35–40). Those basic principles focus on contact strategies (24–28), empathy creation (29–32) and building efficacy by increasing knowledge and skills (27, 33–38) for reducing stigma by: (1) increasing awareness and understanding of the concrete forms stigma takes, as health workers (or people) are often unaware that they are stigmatizing; (2) addressing the specific fears surrounding HIV transmission that drive care or treatment avoidance behaviors; and (3) working on the negative attitudes that lead to blaming and shaming by health staff who judge people living with HIV to be engaging in socially “unacceptable” behaviors or belonging to groups that are considered “lesser or other” than mainstream society. The pervasive presence of these drivers across diverse contexts is well documented, including in India (3, 8, 12, 15, 20, 23, 40–44). In particular, misconceptions about HIV transmission, blame, and negative attitudes toward people living with HIV have been identified as drivers of stigma among health care professionals in India (8, 15, 16, 40–42).

A key strategy for addressing the drivers of stigma has been participatory training with health facility staff, generally with the delivery of up to 2 days of in-person training (19), with at least one session led by people living with HIV or people from groups affected by or associated with HIV (21). While this approach is well received, and has shown positive results, two key challenges in a busy health delivery system are time and ability to scale up time-intensive programs. Ideally, all staff in a facility, from cleaners to senior doctors, would receive in-person, participatory stigma-reduction training covering all the key drivers of stigma as a standard practice. In reality, most health facilities will struggle to find the time to offer this type of training to all their staff. In response, the DriSti study, a randomized controlled trial, is currently testing a blended-learning HIV stigma-reduction approach, through tablet computer-administered and in-person sessions with nursing students and ward staff in India. This paper describes the process and development of the intervention, and discusses practical lessons learned. The outcome evaluation results of the intervention will be presented once the evaluation study is complete (July 2019).

## Materials and methods

### Intervention context

This manuscript describes the development process and final intervention content that resulted. The intervention content that resulted from the process descripted in this manuscript is now being tested in a cluster randomized controlled trial among 3,600 nursing students and ward attendants in 26 private for-profit, 10 private not-for-profit institutions in Karnataka state, India. The evaluation results will be published as soon as they are available. Eligible participants in the trial are at least 18 years old, willing and able to participate in the intervention and all assessments. They either have either worked as a ward attendant for at least 1 year (average years of working as a ward attendant at baseline = 11 years, minimum 1 year, maximum 42 years) or are enrolled as a second-year nursing student at one of the collaborating institutions. Second-year and first-semester third-year nursing students are eligible, because they would have started their clinical rotations in the first year, would be trained in standard precautions, and would have significantly increased patient contact in the second year of school. They have a range of 1.5–2.5 years of nursing training at this point in their education.

### Conceptual framework

**Figure 1:**
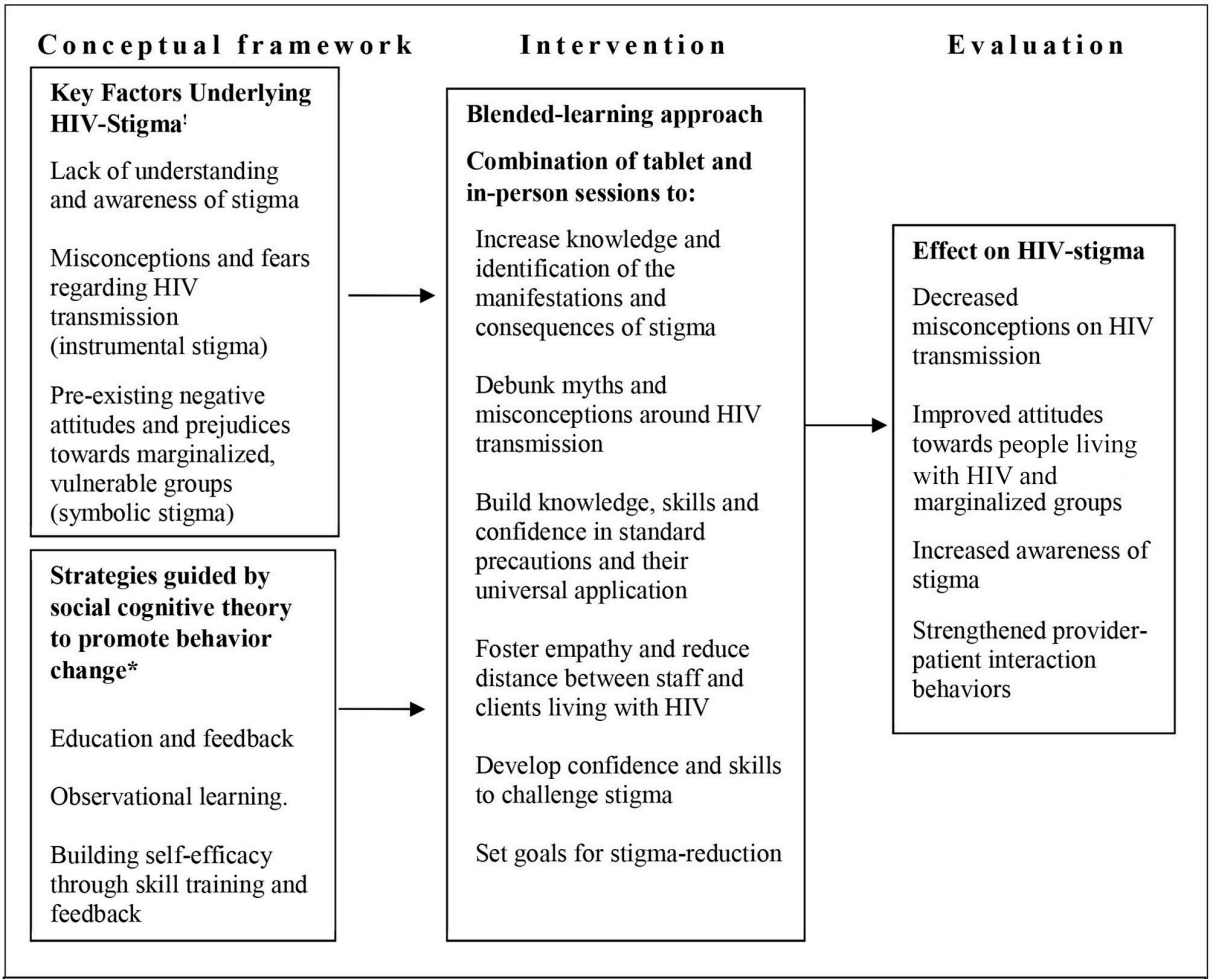
HIV stigma reduction: from conceptual framework to intervention and evaluation. ^!^Factors identified by the “2-factor theory” of HIV stigma and in prior research. ^*^We have drawn upon SCT (33) to identify strategies that promote behavior change. Thus, activities such as computer-based self-tests, patient interaction skills training, interactive games, and PLHIV presentations are designed to educate, promote learning, and build self-efficacy and skills; key elements of behavior change as conceptualized by SCT (34).

The development of the intervention for the DriSti study was guided by a conceptual framework (Figure 1) that is grounded in behavioral change strategies. The behavior change strategies were based on social cognitive theory principles (33) that stress the importance of combining interpersonal interactions with specific strategies that promote behavior change. This can include creating safe spaces for contact between the perpetrators of stigma and the stigmatized (contact strategies) (24–28) which works to break down differences and foster empathy (29–32). It can also include observational learning and role play with feedback to promote self-efficacy. Thus, activities such as computer-based self-tests, patient interaction skills training, interactive games, and presentations by people living with HIV were designed to educate, promote learning, reduce distance and the “us vs. them,” create empathy and build self-efficacy and skills—all of which are key elements of behavior change as conceptualized by social cognitive theory (34). These strategies were then used to develop activities to address the key actionable drivers of stigma: fears and misconceptions surrounding HIV transmission (i.e., “instrumental stigma”); negative attitudes toward people living with HIV and marginalized groups vulnerable to HIV infection (i.e., “symbolic stigma”) (45–48); and lack of awareness of stigma and its effects (1). In addition, the development was grounded in the Indian context and based on previous research in health care settings in India (8, 14–16, 20, 40, 41, 49–51). The team developing the intervention comprised of Indian experts residing in India, as well as US experts with years of experience working in India, with combined expertise in infection control and standard precautions, stigma and stigma-reduction, behavior change, delivery of HIV and other medical services and most importantly the lived experience of people living with HIV in India.

**Figure 2:**
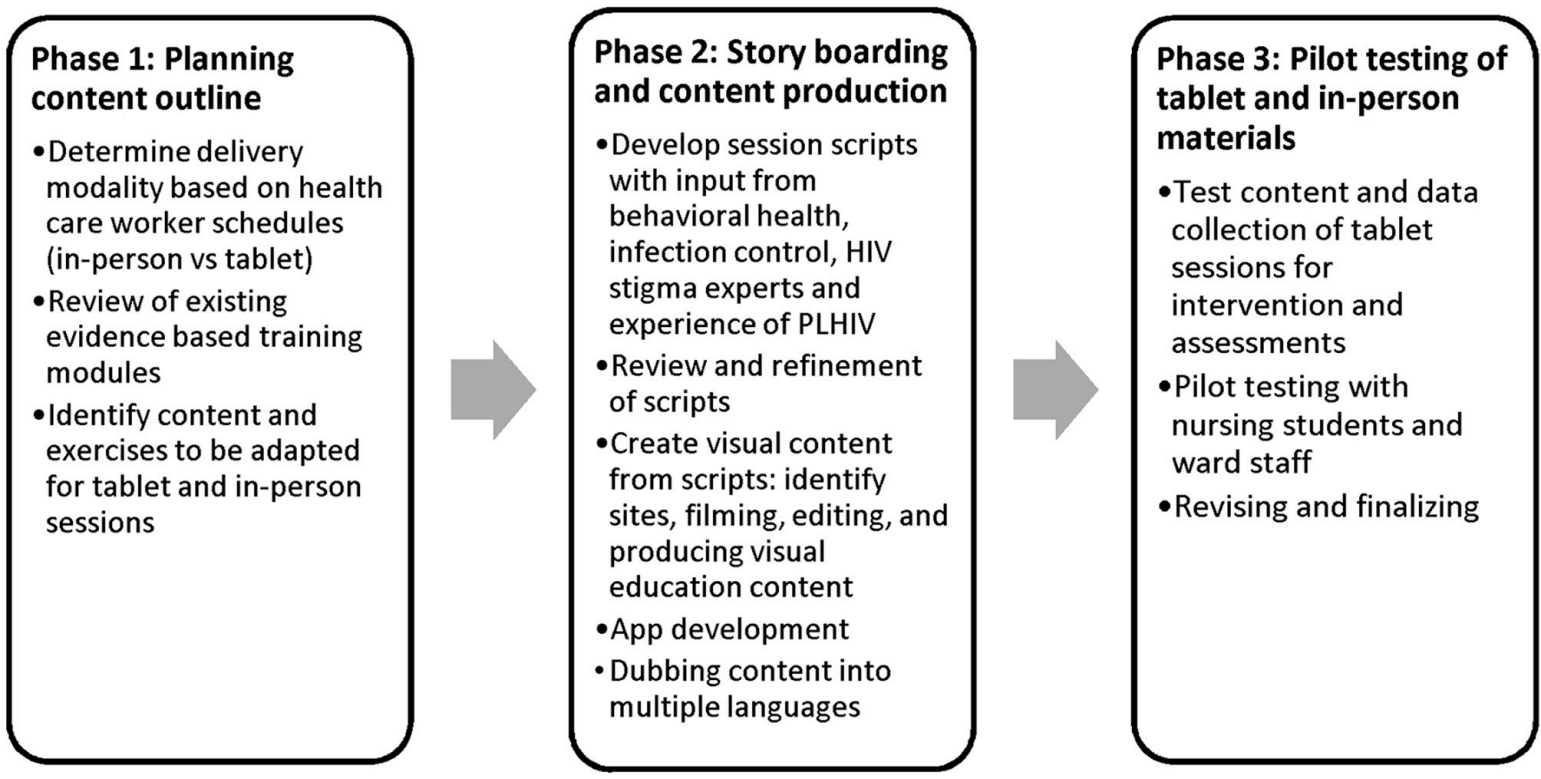
DriSti developmental road map.

Four key practical considerations guided the design and content development of the intervention for the DriSti study: (1) the reality of busy health workers' schedules, which constrained time for training, and therefore health workers' short attention spans; (2) the importance of addressing the actionable drivers of stigma (11, 15); (3) the known value of behavior change strategies to create contact between stigmatizer and stigmatized to break down stigma (11, 24, 27) and create empathy (29–32); and (4) building on existing, proven stigma-reduction training materials (49). With these considerations in mind, the intervention design team grappled with two key questions: (1) What is the best delivery modality? and (2) What is the minimal (quickest) intervention package that can be delivered to adequately cover all the actionable drivers of stigma? The delivery modality, intervention methods, and content development were guided by previous work conducted in India (11, 41, 49, 52) and so fully grounded in the Indian context. Development of the intervention content proceeded through three phases (Figure 2): (1) planning content; (2) story boarding—script development and tablet content production; and (3) pilot testing of tablet and in-person session materials.

### Phase 1: building on and adapting existing materials

#### Delivery modality

The first step in the intervention development process was to consider different intervention delivery options (e.g., in-person only, self-directed learning only, or a combination).Two key considerations guided the final decision on the delivery modality: (1) the need for flexibility in the timing and administration of training to mesh with busy health worker schedules, and (2) the demonstrated value of in-person participatory trainings, as well as contact strategies, to reduce stigma and foster empathy (24, 27, 29, 30, 41). Our pilot study with nursing students in Bangalore, India, using in-person sessions, demonstrated the acceptability and feasibility of the sessions to the Indian context (41). With respect to the former consideration, the availability and growing comfort levels with information technology for many populations, along with evidence for the potential for technology-driven self-directed learning, pointed to the utility of an e-learning platform (53–57). At the same time, previous stigma-reduction work has shown the importance of the in-person approach, in particular creating safe spaces for contact between health providers and clients living with HIV outside of the usual provider-client interaction (22, 39, 58). Given this, the way forward pointed to a blended-learning approach that combined self-directed learning through computer administered sessions and in-person learning (see Results section).

With respect to the self or e-learning part of this approach, we chose tablets rather than an online modality because of their mobility. They could be easily brought to the participants in their facilities, did not require access to computers or an internet connection (so did not limit which facilities could receive training) and participants could pick up tablets and sit wherever they could find a quiet corner to work through the training. In addition, as this was a research study, there was an added value in that the tablets collected data that could then be uploaded securely to the data server. Details on the technological requirements and technical aspects of the tablet-based modules are described elsewhere (59).

#### Content development

##### Review of available training materials

The starting point for the content development process was a review of existing stigma-reduction training modules (49, 60), taking into consideration results from an intial pilot adapation of these tools for a fully in-person stigma-reduction training with nurses in India (41), as well as the behavioral change strategies discussed above. This review assessed what in-person activities might be adaptable to a self-directed learning process on a tablet for each of the three actionable stigma drivers—awareness of stigma, fear (instrumental stigma), and attitudes (symbolic stigma). Through this review, we also hoped to identify what activities were not adaptable to tablet-based learning but were important to include and therefore should be considered for in-person sessions. Once an initial potential set of exercises was identified, these were then taken into consideration against available (realistic) timing for both tablet and in-person learning, to arrive at what the team assessed as the minimal content needed to adequately cover all three stigma drivers and balance the dosage of self (tablet) vs. in-person learning (see results section below). Below is a summary, by key actionable driver, of the types of available activities that were reviewed and considered for inclusion in the intervention.

##### Actionable driver 1: awareness

Training modules available for our review focused on helping participants concretely learn what stigma is and what its consequences are (i.e., how stigma fuels the HIV epidemic), as often we are unaware that what we are doing is stigmatizing. Specifically, the available training materials focused on creating understanding of what behaviors, language, and attitudes are stigmatizing in general, but also particularly in the health facility setting. Materials reviewed also focused on the effects of stigma on the well-being of the individual client and the HIV epidemic more broadly.

Existing participatory in-person training materials offered several exercises to address this driver. These included a range of different ways to prompt participants to name or identify stigma on their own, by describing what is happening in different pictorial scenarios or why it is happening and the consequences. Another way is by taking a “stigma walk” through a health facility and mapping all the places in a health facility where stigma happens, describing the forms of stigma that occur in a particular location, why they happen, and the effects on clients and staff. This could be done either through drawing a map and marking where stigma happens and what it looks like, or physically walking through the facility and doing the same. Additional exercises deepened understanding with further analysis of the causes, forms, and effects of stigma (e.g., through a problem tree), as well as of secondary stigma feared or experienced by health providers themselves and how this could shape service delivery behavior.

##### Actionable driver 2: fear of HIV transmission (instrumental stigma)

Reviewed exercises addressed fear of HIV transmission through three approaches: (1) identifying and addressing knowledge gaps about HIV transmission; (2) focusing on how to reduce real risk when delivering services (standard precautions); which was then linked to (3) creating understanding of the importance of universal application of standard precautions and how selective use of standard precautions is stigmatizing. The first approach focused on addressing specific fears participants might have about casual contact with patients living with HIV, as well as more general myths and misconceptions about HIV transmission. It included understanding the root or underlying fear and addressing that directly. For example, fear of touching personal effects or touching the skin of a person living with HIV (e.g., while taking blood pressure) often comes from a belief that HIV can be transmitted through sweat, combined with not understanding that HIV cannot survive outside the body for very long. Available exercises encouraged training participants to share their specific contact fears and explain why they thought HIV could be transmitted through that type of contact, and then helped them understand why HIV cannot be transmitted that way. To deepen this understanding, follow-on exercises had participants practicing their newly acquired information by explaining to other participants why HIV could or could not be transmitted in certain ways.

The second and third approaches are linked and acknowledge that health workers do face real risks of HIV transmission in the course of their work, for example from needle sticks. However, they often respond to these risks in unnecessary ways that are stigmatizing and lead to inadvertent disclosure of a patient's HIV status. Using two pairs of gloves or selectively using gloves only with patients who are known or suspected to be living with HIV for procedures that require use with all patients (e.g., taking blood), or for procedures that do not require gloves (e.g., taking blood pressure), stigmatizes those patients. In our review of existing training materials, response to these two linked issues was through: (1) better knowledge and application of standard precautions (often referred to as universal precautions); (2) emphasis on the importance of applying standard precautions with all patients and not just patients known or assumed to be living with HIV; (3) the purpose and availability of post-exposure prophylaxis in case of an accidental exposure to HIV (e.g., a needle stick); and (4) how selective application of standard precautions is stigmatizing and leads to HIV-status disclosure.

##### Actionable driver 3: attitudes (symbolic stigma)

Reviewed exercises focused on helping training participants recognize attitudes that are stigmatizing and how these can influence the quality of care delivered, often in unrecognized ways. For example, those attitudes can be expressed in verbal and body language, through shaming, blaming, and judgment, as well as in other discriminatory practices like making certain patients wait to be seen last, even if they had arrived earlier than others. Available exercises guided participants through participatory processes to identify individuals or groups who are stigmatized by society and the ways in which they are stigmatized. One exercise asked participants to list all the names they have heard used toward the groups they have identified as stigmatized, the way these groups are treated and why this happens, and how it makes the people on the receiving end feel. This led to a second set of exercises that focused on building empathy, for example through self-reflection exercises, and on breaking down distance between stigmatizer and stigmatized by creating safe spaces for interaction. A commonly described way to provide this opportunity is through testimonials or interactive panels with people who are living with HIV or experiencing stigma for other reasons, or by having co-trainers throughout the training who are living with HIV or belong to other stigmatized groups.

### Phase 2: script development and tablet content production

Once the review of the existing exercises was completed, the study team determined which in-person exercises could be “translated” for administration on a tablet, the minimal content needed to cover the three drivers of stigma, and given the busy schedules of health workers, a realistic time needed to deliver the intervention. With these considerations in mind, the team arrived at an overall intervention strategy of two tablet sessions (roughly 1 h each) and one in-person session (1.5 h). Next, we outlined which types of activities would cover each driver and which could be administered using the tablet vs. through the in-person session. Consideration was also given for how the sessions would build on each other and reinforce the previous session. Table 1 summarizes how the three drivers are covered across the three sessions. More in-depth detail on final session content is provided below in the results section.

**Table 1:** Summary table: drivers of stigma targeted by each session.

**Drivers of stigma**	**Tablet session 1**	**Tablet session 2**	**Group session 3**
Awareness	X	X	X
Fear of HIV transmission (instrumental stigma) and lack of faith in standard precautions		X	X
Attitudes (symbolic stigma)	X		X

Once example exercises were selected and available session times agreed upon, draft scripts were written through a team effort that brought together Indian and US-based experts in infection control and standard precautions, stigma-reduction experts, behavioral scientists, and the experience of people living with HIV in India. Focusing on the three stigma drivers, topics and storylines to address awareness, fear, and attitudes were identified and scripted, then reviewed by a team that included health professionals (both associated with the study and external experts) and people living with HIV. The scripts went through several rounds of review and refinement, paying special attention to ensure they were appropriate to the Indian context.

The next step was turning the scripts into visual content for the tablets, specifically short video films. This required assembling and managing a diverse team that included staff with skills to produce the visual educational content, deliver the content (actors), set up scenes with appropriate props, and deliver on the information technology aspects, such as transferring and formatting the video for tablet viewing, for example. This latter aspect is covered in detail elsewhere (59), so the focus here is on the filming and production side of developing the tablet content.

The first step in the filming was to engage the actors. Initially, the plan had been to engage only professional actors; however, given the continuing pervasive stigma surrounding HIV and people living with HIV, it was difficult to find professional actors who would agree to play the roles of persons living with HIV. Some even had issues being in scenes with people living with HIV, for fear of being identified as someone living with HIV. For example, one actor refused to portray the role of a family member sitting beside the person living with HIV in the hospital waiting area, once the first practice shot was over. The director's initial response to this challenge was to blur the faces of all characters so that the professional actors would agree to play these roles. Given the main purpose of the project is to break down stigma, we thought that this response was unacceptable. In the end, the project team reached out to contacts within the network of people living with HIV in Karnataka State who are publicly open about their HIV status to find some who agreed to portray health care clients living with HIV. In addition, given the health facility setting and the need to depict certain actions correctly for the standard precautions clips, nursing students and medical staff were also recruited to participate.

The next step in the filming was setting the scenes. Filming was completed through a mix of created staging and filming in working hospital departments. A hospital had a new wing that was not yet in use, which allowed the team to use that space to create many of the different scenes. This made filming easier and more efficient. It, however, did require significant attention to detail, to make sure the scenes were correctly staged and appeared realistic. For example, treatment center signage, patient information posters, and hospital notice board props were created and placed in appropriate locations. At times, there was a need to borrow real props for the recreated scenes, including items like nurses' and doctors' attire, patient gowns, hospital linen for the patient beds, actual antiretroviral therapy pill bottles, and the green treatment books provided by the antiretroviral therapy clinic and carried by patients to their appointments.

Scenes that had to be shot in departments where clients were waiting, for example the pharmacy, required additional considerations. Permission had to be sought from the hospital and departmental management, and waiting clients had to be continuously managed as they became uneasy with the process, the lights, and the inquisitive crowds, and new clients had to be oriented as they entered the room.

For both types of filming location, it was important to have experts review the staging to ensure it was correct, as well as make sure the actors were demonstrating the procedures correctly. That included having a standard precautions expert come in to watch and advise on the scenes demonstrating standard precautions. Having nursing students play nurses in the videos also helped, as they were already knowledgeable of how to hold a syringe, put on gloves, etc. Language also posed some technical challenges. The filming was primarily done in English, with dubbing in two other Indian languages—Kannada and Hindi. The dubbing turned out to be a challenge as the languages were different enough to pose lip syncing issues.

### Phase 3: pilot testing

Once the tablet content was ready, tablet devices were tested for the flow of the baseline assessment, the intervention, and the post-intervention assessment modules. Data collected from the tablet devices were transferred to a central research database wirelessly through the internet and validated for accuracy. Once the tablets were ready, they were handed over to the study team for pilot testing with 10 nursing students and 10 ward staff. Of the 20 participants who participated in the pilot study, 9 nursing students and 9 ward staff also underwent pilot group sessions.

Feedback from the participants was collected in the third, in-person, session. Participants were asked to reflect on what they had learned from the tablet sessions by sharing in pairs, and then reporting back to the group on what the other person had shared. The feedback on learning from the tablet sessions and the in-person session confirmed that the content was meeting the session objectives. For example, participants described—to their session partners and directly—being unaware that stigma existed, of the forms it can take, and of who the recipients are: “*We could discriminate against patients unknowingly,”* said a nursing student in the pilot test. They also talked about acquiring new knowledge about HIV transmission and how that reduced fear of HIV and clients living with HIV. “*First of all, she was very scared about HIV but now after seeing the video shown by you, she got to know that, by eating with HIV patients or by hand shake HIV does not get transmitted*,” a ward staff reported of her partner in the pilot test. This was linked to better understanding of standard precautions and the need to apply them universally: “*She used to wear double gloves for patients living with HIV, but now she understood that single gloves are enough*,” a nursing student reported of her partner in the pilot test. At the end of the in-person pilot session, which included testimony by a person living with HIV, one of the nursing students shared the following: “*In my life during the first year of studies I did not go to the side of patients with HIV, because I was so scared to go there. Now I feel very bad for having done that. I could have shown them empathy as a health professional.”* Overall feedback at the end of the three sessions indicated that participants found the training informative and a positive experience.

Two key lessons were learned from pilot-testing the tablets. The first was the need to provide headphones so that the sessions could be listened to in private. The second was that one cannot assume the participants will be comfortable going through the sessions on their own; it is necessary to have a team member present while they are doing the sessions, so that they can answer questions as they progress.

## Results

Our key result is the final design and content of the blended-learning intervention package to reduce HIV stigma and practical lessons learned in the process of development.

### Final intervention package

The final curriculum for the intervention was condensed into three sessions with a total of eight modules; the first two sessions are self-administered by a tablet computer, and the third session is conducted in a small group of approximately 15 participants. Sessions should be scheduled 1 week apart. Participants can choose to complete each tablet session in English, Hindi, or Kannada, with the option to change to their language of choice during the session. A study team member administers the tablet session, explains the process, and is present throughout the tablet session administration to answer any questions the participants might have. The sessions and modules are described below.

#### Session one (tablet, 50–70 min)

The first tablet session consists of four separate modules and focuses on describing the concept of stigma and building awareness of stigma in general, as well as beginning to form an understanding of stigmatizing attitudes (Table 2). Each module is interspersed with commentary by a narrator who not only provides instructions to the exercises, but also asks questions, pauses to give the participant time to reflect, and then summarizes and reinforces key learning points at the end of each module and again at the conclusion of the session.

**Table 2:** Summary of tablet session 1: describing the concept of HIV-related stigma.

**Drivers of stigma**	**Learning activities**
Awareness	Video of stigma experienced by a person living with HIV in the household setting Defining stigma: participants write their own definition or understanding of stigma Identify stigmatized groups and non-stigmatized groups from a selection of pictures; video feedback for each picture explains why certain groups are stigmatized Narrator and colleague discuss the drivers of stigma, the concept of layered stigma, labeling and consequences of stigma Self-reflection on stigmatizing attitudes A virtual participatory walk-through of a hospital explores where and how stigmatizing attitudes and discriminatory practices may occur Interactive multiple-choice questionnaire testing understanding of HIV-related stigma Rating of agreement or disagreement with statements about people living with HIV to explore beliefs and attitudes; narrator defines stereotypes, where they come from and their consequences
Attitudes	Self-reflection exercise asking participants to identify and consider a time in their life when they have experienced stigma or discrimination and how it made them feel After each video selected during the virtual video walk-through, the narrator asks the participant to reflect on, “How would you feel if this happened to you?” “What kind of stigma did you see?” and “Why did health care workers/patients stigmatize?” Three video testimonials by people living with HIV accompanied with self-reflection questions to build empathy and reduce blame

*Module 1:* The first module aims to define stigma and begins with an introductory video portraying stigma in a household setting. The narrator asks the participant to reflect and record their definition of stigma. After allotting participants 60 s, the narrator returns to provide a definition of stigma and discrimination and provides some examples of potentially stigmatizing actions. Next, the participants engage in an interactive exercise in which they are shown pictures of different groups of people and asked to identify the groups they think are stigmatized. The narrator has a discussion with her colleague in the module video and asks her to describe what happens when we stigmatize and the consequences of stigma for people living with HIV. The participants are then asked to reflect on a time when they experienced stigma and to identify or recognize any possible stigmatizing attitudes they may hold.

*Module 2:* The participant engages in an interactive virtual walk-through in the hospital to identify how different forms of stigma occur in different settings. In the walk-through, participants are presented with a virtual layout of a hospital depicting the various departments where patients interact directly with health care providers. The participant is asked to identify specific locations in the hospital where they think stigma may occur for clients living with HIV. Clicking on the virtual map allows the participant to have a 360° view of each location of their choice and view a short video showing a person living with HIV experiencing stigmatizing attitudes and discriminatory practices specific to that location. The virtual map captures two in-patient (in-patient ward and maternity ward) and 12 out-patient locations (the registration counter, out-patient waiting area, physician's room, blood bank, pharmacy, emergency room, operating room, antenatal clinic, hemodialysis, integrated counseling and testing center, antiretroviral therapy center, or a government hospital out-patient department). At the end of the video presentation, the participant is asked to reflect on how they would feel in this situation by answering multiple choice questions. The participant must view a minimum of three out-patient and one in-patient location videos before they can proceed to the next exercise.

*Module 3:* The participants explore their beliefs and attitudes about people living with HIV and other marginalized groups by taking a survey. Participants select if they strongly agree, agree, disagree, or strongly disagree with a statement such as, “Nurses have a duty to inform the spouse and family of a person who is living with HIV.” The narrator provides feedback by describing the concepts and effects of stereotyping, judgment, and empathy.

*Module 4: Three* video testimonials are presented by persons living with HIV describing their experience with stigma in a health care setting and elsewhere. The first part of each testimonial begins with how and where the person came to learn about his or her diagnosis. The narrator then asks the participants to reflect on how they would feel if they were in this situation and what actions they would have taken. The second half of the testimonial describes the effect of HIV-related stigma in the lives of people living with HIV.

#### Session two (tablet, 30–45 min)

This session has four modules designed to reduce instrumental (fear-driven) stigma by increasing knowledge of HIV transmission, correcting transmission misconceptions, building understanding of how fears of HIV transmission influence how health workers provide care and of the importance of implementing standard precautions with all patients, regardless of HIV status (Table 3). Like session one, this session uses narrator commentary to provide instructions, ask self-reflective questions and revisit key learning points for each module.

**Table 3:** Summary of tablet session 2: reducing instrumental (fear-based) stigma.

**Drivers of stigma**	**Learning activities**
Awareness	Identifying areas in the hospital from the walk-through in session 1 where stigma may occur
Fear of HIV transmission	Statements about routes of HIV transmission and misconceptions are presented to the participant with accompanying videos explaining why the statement is true or false Identifying level of fear associated with high- and low-risk procedures depicted in a set of four pictures; video of a conversation between health care staff (either two nurses or ward staff) about why a procedure is of low risk or how to use standard precautions to protect against HIV transmission in high-risk situations Videos showing how participants can protect themselves from HIV transmission by using standard precautions

*Module 5:* The first module of session two revisits the key learning points of session one, including the virtual hospital walk-through. The participant is asked to indicate which locations they visited during the virtual walk-through that were the most surprising locations to find stigma.

*Module 6:* This module explores misconceptions about how HIV can be transmitted, including, for example, the belief that injection with a sterilized syringe can transmit HIV or that using the same toilet as someone living with HIV puts one at risk for transmission. For each statement, the participants indicate if a co-worker has told them it is a mode of HIV transmission. A feedback video follows to explain why each statement is true or false—HIV cannot be transmitted using the same toilet as someone living with HIV because the virus cannot survive outside the body, for example.

*Module 7:* The third module of session two explains how fear of transmission may influence the participant's behavior. Four images depict different procedures the participant may perform on patients. These scenarios are tailored to either nursing students (taking an oral temperature, drawing blood, assisting in childbirth (conducting delivery), and bathing a patient at bedside) or ward staff (taking oral temperature, dressing a sore, bathing a patient at bedside, and making the bed). The participant then selects the severity of fear (no fear, mild fear, moderate fear, severe fear) they experience when performing the procedure on a person living with HIV. For each picture scenario, a video shows two nurses or two ward attendants having a conversation with each other. One states they are fearful of performing the procedure in the picture, while the other explains why the procedure is low risk or how to use standard precaution to protect against transmission. The narrator then reinforces the consequences of HIV-related stigma.

*Module 8:* The last module of session two focuses on standard precaution using a series of four videos. The narrator begins the session by explaining the importance of using standard precautions for all patients, regardless of their HIV status. Each video contains two scenarios. The first is an excerpt from session one and shows mistakes health care workers may make while working, which could increase the transmission risk of an infectious disease. It also demonstrates that “excessive” precautions can be stigmatizing. The second scenario shows the correct set of practices to be applied in the situation. The narrator reinforces the importance of using standard precautions with all patients to reduce risk of acquiring HIV or other infectious diseases.

#### Session three (in-person, group session, 90 min)

The group session focuses on patient interaction skills and is co-facilitated by the intervention staff and a person living with HIV (Table 4). The session begins with a discussion of the participants' experiences with the tablet sessions and a recap of the key learning points from those sessions. To begin the discussion and facilitate an open sharing environment, the co-facilitator living with HIV describes how they learned about their HIV status, their reactions to learning they were living with HIV, and the process of disclosing the diagnosis to family members. The facilitator then describes a stigmatizing situation encountered at a hospital, followed by a positive health care encounter (**Box 1**). The facilitator provides feedback on how health professionals can provide clients living with HIV with non-stigmatizing supportive care. The group then divides into smaller groups for role-playing exercises of hospital-based scenarios depicting where stigma could occur, such as when drawing blood or during admission and discharge. Each group will be assigned a scenario to role play that will demonstrate stigmatizing and non-stigmatizing behaviors when handling situations. Scenarios depict ways a client living with HIV could experience stigma or discrimination in a health care facility (e.g., intake, blood draw, sheet change). The small groups practice their scenario and then perform their scenario for a larger group, demonstrating both stigmatizing and non-stigmatizing behavior. Feedback is provided by the facilitator living with HIV and other group members, who discuss the scenarios and how and why stigma happens, the consequences of stigma for patients and health care workers, and how health care workers can reduce and avoid stigmatizing behaviors. The session concludes with participants writing down one thing they will commit to do to reduce stigma in health care facilities toward people living with HIV and sharing this commitment with the group.

**Table 4:** Summary of group session 3.

**Drivers of stigma**	**Learning activities**
Awareness	Discussion recapping the tablet sessions identifies new knowledge, insights and stigmatizing behaviors Skills-building through role-playing scenarios in the hospital where stigma may occur
Fear of HIV transmission	Skills-building through role-playing scenarios in the hospital where stigma may occur
Attitudes (e.g., blame, judgment, shaming)	Safe discussion space co-facilitated by a person living with HIV Co-facilitator living with HIV shares testimonial of experiences with diagnosis and family's reaction to disclosure, as well as both negative and positive interactions with health care workers Advice on how to provide non-stigmatizing care to clients living with HIV

Box 1 **Synopsis of a personal story shared by the co-facilitator living with HIV.**

- The circumstances—when and how—they found out they were living with HIV
- Their experience disclosing their status to their family, and how the family reacted
- Details of a stigmatizing or discriminatory experience at a health facility
- Details of a positive experience at a health facility.

## Discussion

The importance of addressing HIV stigma in health facilities to improve quality of care and patient outcomes is well documented and accepted (18, 20–23). What is less well understood is how to effectively reduce that stigma within the confines of an extremely busy health delivery system and limited health facility staff time for participation in training or other learning activities. The three-session intervention designed for the DriSti study offers a potential solution to this challenge, combining two self-directed, tablet-administered learning sessions with one in-person group session. While it will be sometime before the trial is completed and the results known, feedback from early implementation experience suggests that such an approach is feasible and well received by both nursing students and ward staff. Most participants have actively engaged in the sessions and provided positive feedback through comments about the training and the learning process. As there was a time gap between the completion of the tablet sessions and the group session, the intervention coordinator noted that several participants told the facilitators that on completion of the videos (tablet sessions), they changed their behavior toward clients living with HIV because “*they have removed that fear from our mind*.”

It should also be noted that since the content for this intervention was designed, the field of mHealth has progressed significantly (61–65). Therefore, the range of options for delivering the self-directed learning component is rapidly increasing. For example, the tablet session of the intervention could now be delivered on a smart phone, tablet device, or through a computer terminal in the ward with cloud access to content or a learning management system.

In the process of developing the three-session intervention there has been some important learning. Key among them is the breadth of expertise that must come together to design and produce stigma-reduction intervention content for a blended-learning approach. The DriSti study had to assemble and manage a diverse team with complementary expertise and skills in stigma-reduction and behavioral change, information technology, film direction, and standard precautions. The team also learned not to underestimate the challenges of finding appropriate space for filming—whether staged or in working departments located within a hospital. This included obtaining the necessary permissions to use that space and managing the filming while staff and clients were present, as well as the intricacies of creating appropriate settings for staged scenes not filmed in real settings.

Another central lesson was not to underestimate how the topic of the scenes—stigma toward people living with HIV—could be a challenge itself to developing stigma-reduction material. While all professional and non-professional actors went through a consent process at the start to ensure they understood what the video was about, that paid professional actors would hesitate to portray a family member of a person living with HIV, let alone a person living with HIV, underscores not only the prevalence and intensity of HIV stigma in the community, but also the importance of HIV stigma-reduction interventions. On the flip side, the assembling of such a diverse team to produce this material in and of itself provided an opportunity for stigma-reduction. The diverse range of skills needed—acting, directing, and information technology—to produce the content opened spaces for dialogue and learning on HIV and stigma with groups of people who would not typically engage on this topic.

Most important is the lesson that people with the lived experience of HIV and stigma must be central to the design and delivery of HIV-stigma reduction interventions. People living with HIV provided input into the script development and participated in the videos portraying the roles of people living with HIV. The in-person group session revolves around having a person living with HIV present to share experiences and interact with participants. Experience to date indicates this is the single most important interaction of the intervention for participants. It is typically the first time they have interacted with an openly positive person living with HIV outside a health facility or patient/provider setting. As has been shown in reducing stigma related to mental health, contact between stigmatizer and stigmatized is central to effective stigma reduction (27).

While this blended-learning approach was designed specifically to address the busy time-constraints of health workers, of note is that even at three relatively brief sessions, scheduling and time availability remained a challenge for ward staff working in the hospital. Ward staff have much less time than the nursing students, and scheduling them for any of the sessions, particularly gathering enough of them for the in-person group session, is a challenge at times. For ward staff who work night shifts, sessions must be delivered early in the morning before staff leave the facility, which can be a sub-optimal time for learning. Interestingly, once the ward staff begin the first tablet session, most are “hooked” and want to know more. While the script for the in-person session is a concise 1.5 h, the study team noted that for ward staff, the in-person session may need to be shortened to an hour. One way to work within a 1-h window and ensure all aspects could be covered would be to have smaller groups of ward staff (8–10 participants) per session. Another option may be to break this session up into two shorter sessions. On the other hand, many of the in-person sessions for nursing students run up to 2 h because the students have more free time available, and therefore can spend more time in discussion. Another difference between the two groups is in comprehension of the activities, with ward staff generally needing more explanation than nursing students, especially for the role-playing exercise. Ideally, facilities should provide staff with time to complete the training during working hours.

Lastly, there are some limitations to the approach. Some of the participants noted that their supervisors or senior colleagues are stigmatizing and discriminating, indicating the need to move this intervention beyond ward staff and nursing students to all levels of health facility staff. This approach, training all levels of staff, is currently being implemented in Thailand (66) and Ghana, Jamaica, and Tanzania (67). In addition, this curriculum is designed to address only one level within health facilities—the individual or interpersonal. Intervening at both this individual and broader institutional level will be important to create sustainable change throughout a health system. This particular study was designed to test an intervention targeted at the individual level, which if successful, can then be combined with other interventions targeting institutional structures, policies and practices.

## Conclusion

The process of developing the DriSti intervention not only offers practical lessons for others who might be embarking on designing their own blended-learning packages for HIV or other stigmas but demonstrates that blended learning is a feasible solution to addressing HIV stigma with time-constrained health workers. We anticipate that as individual health workers are trained in stigma-reduction they will lead by example in their health facilities, challenge stigma as it happens and teach their colleagues how to provide non-stigmatizing care. The content of this intervention is based on materials and experiences in India (41), as well as from around the region and beyond, and similar efforts are underway in Thailand (66) Viet Nam (68), Ghana, Jamaica, and Thailand (67). This underscores the utility of this intervention to change the social norms and behaviors of health workers, as well as potential to generalize or adapt this intervention to other settings in the region and beyond.

## Ethics statement

This study received approval from the Institutional Ethical Review Board of St. John's Medical College and Hospital and the Committee on Human Subjects Research of the University of California, San Francisco. All subjects have given written informed consent in accordance with the Declaration of Helsinki.

## Author contributions

LN conceptualized and drafted the paper, ME and AM contributed to drafting the paper. LN, ME, KS, TR contributed substantially to the conception and design of the study and intervention and contributed to draft revisions. TR, DD, KR developed the tablet-based session. DP was the intervention coordinator and provided details on intervention implementation. All authors reviewed the manuscript.

### Conflict of interest statement

The authors declare that the research was conducted in the absence of any commercial or financial relationships that could be construed as a potential conflict of interest.
